# Ketoconazole-Fumaric Acid Pharmaceutical Cocrystal: From Formulation Design for Bioavailability Improvement to Biocompatibility Testing and Antifungal Efficacy Evaluation

**DOI:** 10.3390/ijms252413346

**Published:** 2024-12-12

**Authors:** Ioana Baldea, Remus Moldovan, Andras-Laszlo Nagy, Pompei Bolfa, Roxana Decea, Maria Olimpia Miclaus, Ildiko Lung, Ana Maria Raluca Gherman, Alexandra Sevastre-Berghian, Flavia Adina Martin, Irina Kacso, Vlad Răzniceanu

**Affiliations:** 1 Department of Physiology, “Iuliu Haţieganu” University of Medicine and Pharmacy, 400006 Cluj-Napoca, Romania; baldeaioana@gmail.com (I.B.); remus_ri@yahoo.com (R.M.); roxanadecea@yahoo.com (R.D.); berghian.alexandra@umfcluj.ro (A.S.-B.); razniceanu.vlad@elearn.umfcluj.ro (V.R.); 2 Department of Biomedical Sciences, Ross University School of Veterinary Medicine, Basseterre P.O. Box 334, Saint Kitts and Nevis; anagy@rossvet.edu.kn (A.-L.N.); pompeibolfa@gmail.com (P.B.); 3 National Institute for R&D of Isotopic and Molecular Technologies, 400293 Cluj-Napoca, Romania; maria.miclaus@itim.cj.ro (M.O.M.); ildiko.lung@itim-cj.ro (I.L.); raluca.gherman@itim-cj.ro (A.M.R.G.)

**Keywords:** ketoconazole, fumaric acid, cocrystal, biocompatibility, scale up, liver toxicity, pharmacokinetic profile

## Abstract

Development of cocrystals through crystal engineering is a viable strategy to formulate poorly water-soluble active pharmaceutical ingredients as stable crystalline solid forms with enhanced bioavailability. This study presents a controlled cocrystallization process by cooling for the 1:1 cocrystal of Ketoconazole, an antifungal class II drug with the Fumaric acid coformer. This was successfully set up following the meta-stable zone width determination in acetone–water 4:6 (*V*/*V*) and pure ethanol. Considering the optimal crystallization data, laboratory scale-up processes were carried out at 1 g batch size, efficiently delivering the cocrystal in high yields up to 90% pure and single phase as revealed by powder X-ray diffraction. Biological assays in vitro showed improved viability and oxidative damage of the cocrystal over Ketoconazole on human dermal fibroblasts and hepatocarcinoma cells; in vivo, on Wistar rats, the cocrystal increased oral Ketoconazole bioavailability with transient minor biochemical transaminases increases and without histological liver alterations. Locally on Balb C mice, it induced no epicutaneuous sensitization. A molecular docking study conducted on sterol 14α-demethylase (CYP51) enzyme from the pathogenic yeast *Candida albicans* revealed that the cocrystal interacts more efficiently with the enzyme compared to Ketoconazole, indicating that the coformer enhances the binding affinity of the active ingredient.

## 1. Introduction

The pharmaceutical industry is constantly working on designing new medicinal products with adequate bioavailability regarding the Active Pharmaceutical Ingredient (API) solubility and intestinal permeability to provide an effective and safe treatment [1]. However, at present, up to 40% of APIs formulated as oral delivery drugs exhibit poor aqueous solubility and dissolution rates (class II according to the Biopharmaceutical Classification System, BCS [2]). These are critical aspects that affect the pharmaceutical potential [3]. Various options are available to modulate the physicochemical and pharmacokinetic attributes of the API without altering its chemical identity, such as lipid-based formulations [4], or solid-state forms by rational use of crystal engineering [5]. This approach may conduct to co-amorphous solid dispersions and a variety of crystalline forms, defined as salts, hydrate/solvates, polymorphs, and cocrystals [6]. To date, the salt formulation is the most widely applied strategy focused on the solubility enhancement of ionizable molecules; therefore, almost 50% of drug products are developed in their salt form [7]. Additionally, pharmaceutical cocrystal formulations represent another efficient alternative, aiming to modify and improve the performance of API molecules [6]. Cocrystals are crystalline structures composed of two or more neutral components connected via non-covalent interactions at a precise stoichiometric ratio [8]. To form the pharmaceutical cocrystals, mandatory coformers are required, namely the API and the pharmaceutically acceptable ingredients considered appropriate for use (Generally Recognized as Safe, GRAS) [9]. Industrial research in this area has led to regulatory approval of at least ten cocrystal-based drug products. Several more are in the developing stage [5,10].

Ketoconazole (*cis*-1-acetyl-4-[4-[[(2RS,4SR)-2-(2,4-dichlorophenyl)-2-(1*H*-imidazol-1-ylmethyl)-1,3-dioxolan-4-yl]methoxy]phenyl]piperazine, KTZ) is a lipophilic imidazole derivative, part of BCS class II, which exhibits a high permeability but low aqueous solubility. KTZ was the first orally available azole for the systemic treatment of mycoses [11]. The antifungal properties are based on its ability to antagonize fungal cytochrome P450 enzymes such as lanosterol 14-α-demethylase. This prevents synthesis of ergosterol, an important cell membrane component of the fungi that regulate permeability and structural integrity [12]. Oral KTZ may be beneficial in the treatment of keratitis-ichthyosis-deafness (KID) syndrome [13], mood disorders [14], as well as increasing the bioavailability of drugs subject to intense CYP3A4 metabolization [15]. Topical application is recommended for the treatment of localized and superficial dermatophytic infections such as candidiasis, Malassezia-related seborrheic dermatitis, and tinea versicolor [16]. Although KTZ has proven to be a versatile drug useful for both topical and systemic administration, post-marketing reports of harmful drug interactions, hepatotoxicity, and adrenal insufficiency have led the Committee for Medicinal Products for Human Use (CHMP) and the European Medicines Agency (EMA) to recommend the suspension of KTZ oral usage in 2013, followed by the FDA taking action to restrict KTZ-based systemic treatments [17]. On the other hand, the benefits of the drug outweigh the risks, so it has been used off-label as a second-line systemic therapy for castration-resistant prostate cancer [18]. Moreover, in 2014, the EMA approved KTZ HRA for oral use to treat endogenous Cushing’s syndrome (including paraneoplastic forms [19]), based on its inhibitory effect on adrenal enzymes CYP17A1, CYP11B1, and CYP11B22, whereby it functions as a cortisol synthesis inhibitor. KTZ intervenes in several androgen biosynthesis pathways by reducing CYP17A1 activity, which makes it a useful option for the alleviation of symptoms and increasing survival in cases of advanced prostate cancer [18,20].

Such developments in the KTZ therapeutic uses are an incentive to find and evaluate new formulations that may reduce toxicity, improve efficacy, and increase bioavailability.

In this context, we have previously reported the ability of KTZ to form crystalline forms with dicarboxylic acids in 1:1 stoichiometry, through the crystal engineering approach [21]. This research showed the highest aqueous solubility for the cocrystal obtained with the Fumaric acid coformer, namely a 100-fold solubility increase vs. pure KTZ.

Fumaric acid (*trans*-2-butenedioic acid, FUM) is an organic compound involved in metabolic pathways within human cells [22], and is generally used as a non-toxic food additive intended for human consumption in order to combat the proliferation and activity of harmful microorganisms such as bacteria and yeasts [23,24,25]. FUM and its derivatives have various uses in medicine, mainly in the oral treatment of psoriasis and multiple sclerosis, due to their effective immunomodulatory [26] and antioxidant effects [27]. Other studies have shown that fumarate esters exhibit some anti-inflammatory effects in sarcoidosis, granuloma annulare, necrobiosis lipoidica, and antineoplastic activity in malignant melanoma [28].

Administration of intraperitoneal injections with FUM in ICR mice with systemic *Candida albicans* infections have prolonged survival and prevented one-fifth of deaths [29]. Different oral API formulations were shown to benefit from incorporating FUM. The anti-inflammatory drug dexamethasone presented a greater synergistic effect with FUM, which increased efficiency in the treatment of autoimmune and inflammatory diseases [30], naftopidil solubility was improved in solid dispersion form [31], and the cocrystal with pirfenidone exhibited a sustained-release behavior [32]. Furthermore, transdermal penetration of topical gels containing loxoprofen sodium was increased when FUM was used as an enhancer [33].

In view of the potential health benefits that this cocrystal might have, the purpose of the current study was to further (i) determine the limit of supersaturation up to which the solution of the KTZ and FUM mixture is stable or metastable with respect to nucleation, namely the metastable zone width (MSZW), to allow the setting of optimal parameters for (ii) laboratory scale-up cocrystallization process; (iii) test the biocompatibility in vitro on cell cultures of human skin and liver cells, in view of potential local and systemic applications of the KTZ-FUM and (iv) study the liver toxicity and bioavailability of the substance upon oral administration in vivo on rat animal models, respectively, and epicutaneous skin sensitization on mice models; as well as to (v) evaluate the binding affinity and binding energy with biological molecules by a molecular docking study.

Our results evidenced that cocrystallization represents a successful approach for biocompatibility, oral bioavailability, local anti-inflammatory effects, and anti-fungal efficiency enhancement of the poorly aqueous soluble drug KTZ, which supports further use of the KTZ-FUM cocrystal in clinical studies.

## 2. Results

### 2.1. Controlled Cocrystallization Process by Cooling

The nucleation during the cooling step required for MSZW determination was obtained in only two cases, namely the acetone–water 4:6 (*V*/*V*) mixture and pure ethanol, with a cooling rate of 2 °C min^−1^ and 3 °C min^−1^, respectively (see the experimental protocol, Section 4.2). The experimental conditions of the finalized tests are listed in Table 1, and the unsuccessful experiments are illustrated accordingly in Table S1 (Supplementary Materials).

**Figure 1:**
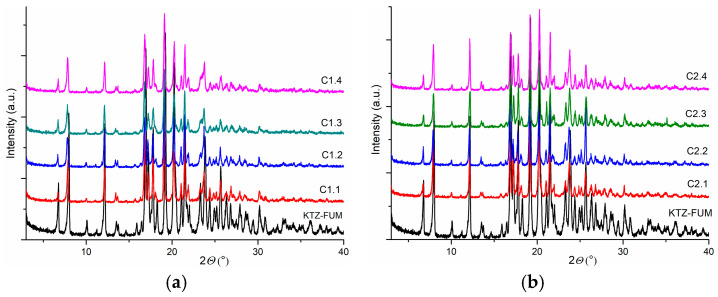
PXRD patterns of KTZ-FUM cocrystal obtained by heating–cooling cycles in (**a**) acetone–water 4:6 (*V*/*V*) and (**b**) ethanol.

The resulting solid samples were subjected to PXRD; for all KTZ tested concentrations, the cocrystal formation as a pure solid form is indicated in Figure 1. The cocrystallization yields were above 70%, and the highest yield value of 85% was recorded for a KTZ concentration of 100 mg mL^−1^ using the acetone–water 4:6 (*V*/*V*) mixture as a solvent.

**Figure 2:**
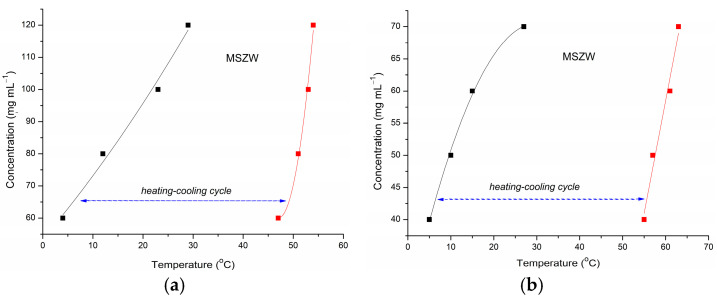
MSZW of KTZ-FUM cocrystal in (**a**) acetone–water 4:6 (*V*/*V*) and in (**b**) ethanol, the clear points (black) and the cloudy points (red).

The parameters for a controlled cocrystallization process of KTZ-FUM were established starting from the MSZW determined in acetone–water 4:6 (*V*/*V*) and in ethanol (Figure 2). The cocrystallization experiment by cooling was designed considering an initial KTZ concentration of 100 mg mL^−1^, for which a wide operating range was observed in MSZW. Thus, complete dissolution of the product did not require a temperature higher than 60 °C. Cooling to room temperature induced nucleation and was enough to obtain a good cocrystallization yield of the cocrystal.

### 2.2. Scale-Up Experiments of the Ketoconazol-Fumaric Acid Cocrystal

The cocrystallization parameters derived within the MSZW experiments were used as indicators for a viable and effective laboratory up-scaling of the cocrystal production to 1 g batch sizes. The first series of scaling tests, namely SU-E1 and SU-E2, were performed applying the method of cocrystallization by cooling in solution using the synthesizer process station Eyela. The reaction conditions are detailed in Table 2.

**Figure 3:**
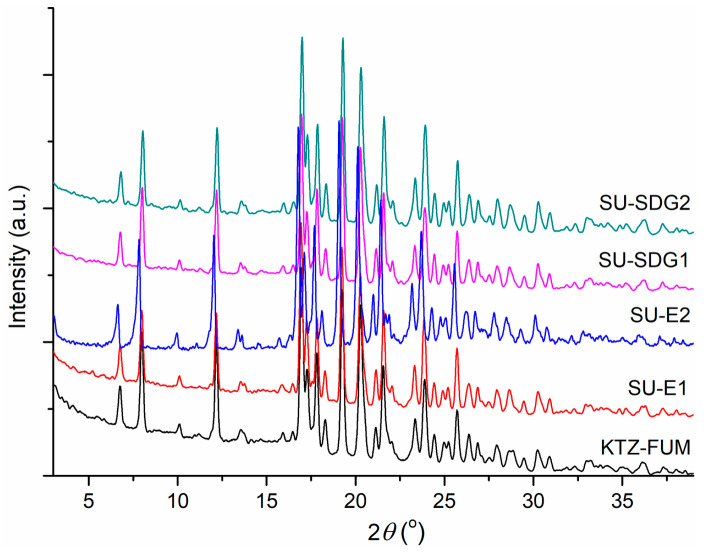
PXRD patterns of the scaled-up experiments of KTZ-FUM cocrystal.

As the PXRD investigation revealed, the KTZ-FUM cocrystal was the only phase obtained in all resulting solid samples (Figure 3) with high scale-up cocrystallization yields, up to around 90%.

The second cocrystallization approach for up-scaling the KTZ-FUM solid form was performed by the mechanochemistry strategy using a ball milling by wet grinding route. Initially, SDG cocrystallization experiments were performed using a reduced quantity of substances and solvents, i.e., 100 mg KTZ and two drops. PXRD analysis confirmed the cocrystal form for each experimental condition, detailed in Table S2 and Figure S1 (see Supplementary Materials). Consequently, the up-scale SDG synthesis based on 1 g KTZ was set up. The reaction conditions, e.g., solvent type/volume, reaction time (Table 3), were enough to yield the cocrystal as a pure form without traces of pure components, according to PXRD (Figure 3).

### 2.3. Biological Assays

#### 2.3.1. Cell Viability

**Figure 4:**
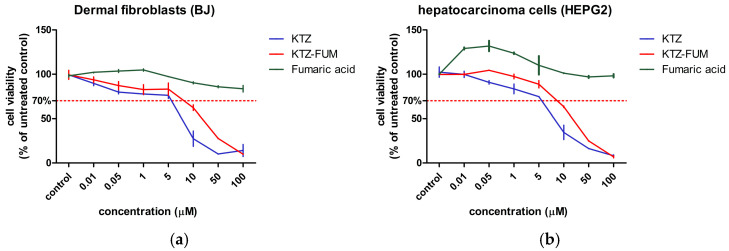
Cell viability of (**a**) dermal fibroblasts and (**b**) hepatocarcinoma cells. Cells were exposed to different concentrations of KTZ, KTZ-FUM cocrystal, and FUM. Data are expressed as % of untreated controls as mean± STDEV (n = 3). A dotted line was drawn at 70%, representing the toxicity limit.

As seen in Figure 4, the cell viability of the BJ and HepG2 was decreased by both KTZ and KTZ-FUM in a dose-dependent manner, while Fumaric acid showed a minimal effect. IC_50_ calculation showed that KTZ-FUM was tolerated better by the cells (Table 4), when compared to the parent drug. In both cultures, Fumaric acid showed a low IC_50_, indicating the inhibition of cell proliferation, despite a small effect on the cell viability as seen in Figure 4.

**Figure 5:**
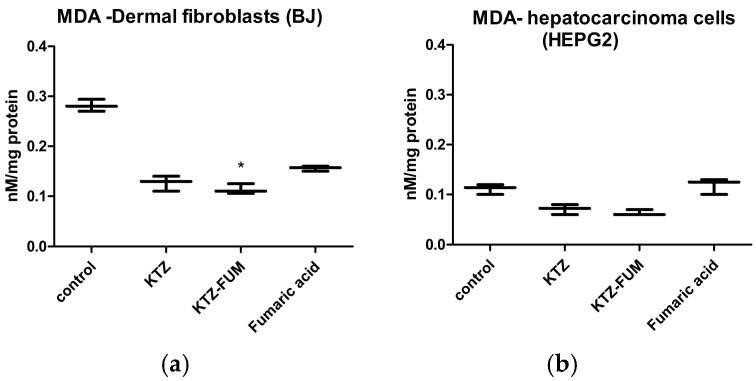
Malondialdehyde—MDA of (**a**) dermal fibroblasts and (**b**) hepatocarcinoma cells. Cells were exposed to KTZ, KTZ-FUM, and FUM in a concentration of 6 µM of each substance. Data are expressed as % of untreated controls as mean± STDEV (n = 3), * = *p* ≤ 0.05.

Oxidative damage induced by cell exposure to the KTZ, KTZ-FUM, and Fumaric acid was evaluated by the measurement of malondialdehyde (MDA), a marker for lipid peroxidation (Figure 5). MDA was decreased by both KTZ and KTZ-FUM. The latter had a more pronounced effect, which was significant in fibroblasts compared to controls. Fumaric acid had a smaller effect in fibroblasts and slightly increased MDA in HepG2, but not significant.

#### 2.3.2. Pharmaco-Dynamic Profile

**Figure 6:**
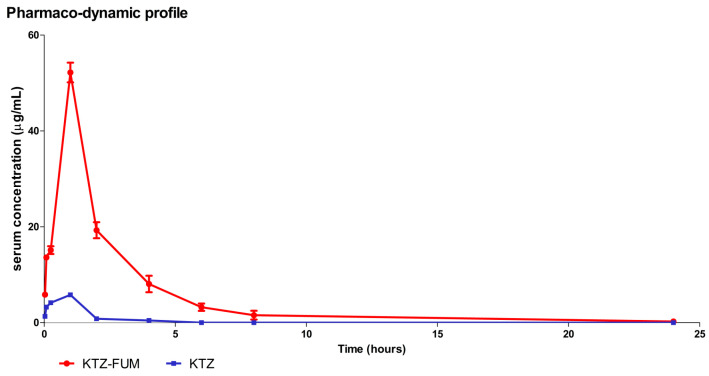
Pharmaco-dynamic profile of KTZ and KTZ-FUM after one oral dose (20 mg kg^−1^ bw) in Wistar rats. Data represent the mean ± STDEV (n = 3).

In our experiments, the relative calculated bioavailability of the KTZ-FUM cocrystal over KTZ, after a unique oral dose, was F = 12.79. This indicates a higher bioavailability of the cocrystal compared to the parent drug. As seen in Figure 6 and Table 5, the peak for KTZ-FUM was reached at 1 h. The maximum concentration KTZ-FUM reached was 8.97 × more than the one for KTZ, which indicates a higher absorption rate from the gut. KTZ-FUM was still detected in plasma at 24 h compared to KTZ, which was not detected after 6 h, indicating that the cocrystal formulation allows for a prolonged release. These data show an improvement in the pharmaco-kinetic profile of the KTZ-FUM cocrystal compared to pure KTZ.

#### 2.3.3. Hepatotoxicity

**Figure 7:**
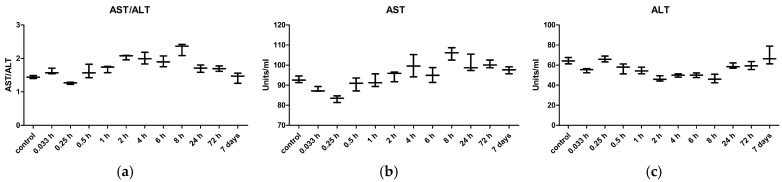
Comparative measurement of transaminases ratio (**a**) AST/ALT, (**b**) AST, and (**c**) ALT from the Wistar rats’ serum after one dose of KTZ-FUM (20 mg kg^−1^ bw), oral administration. Data represent the mean ± STDEV (n = 3).

**Figure 8:**
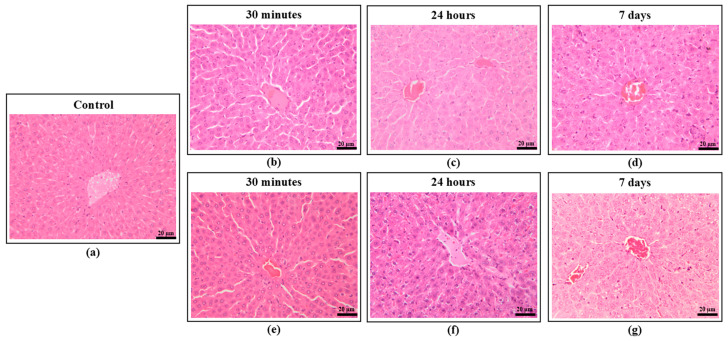
Histopathological images of the liver from the animals exposed to KTZ-FUM for different time points, microphotographs centered on the centrilobular area: (**a**) control, (**b**) KTZ-FUM exposure for 30 min, (**c**) 24 h, (**d**) 7 days, (**e**) KTZ exposure for 30 min, (**f**) 24 h, (**g**) 7 days. All sections show normal hepatic architecture. HE stain, Bar = 20 μm.

Liver injuries induced by a single oral dose of KTZ-FUM (20 mg kg^−1^ bw) were assessed in dynamics by two methods, the biochemical measurement of liver transaminases (AST-aspartate aminotransferase, ALT-alanine transaminase and ratio of AST/ALT) from serum (Figure 7), and the histopathology examination of liver samples (Figure 8). AST, the most important transaminase, decreased in the first 15 min. after the oral administration of KTZ-FUM, then it gradually increased to reach a first peak at 4 h, then a second peak at 8 h; afterwards, it decreased till 7 days, but it remained at a slightly higher level compared to controls. ALT dynamics showed a different trend, with an initial low peak at 15 min, then lower values compared to controls, and then a slow increase until 7 days. The ratio of AST/ALT showed higher values compared to the control until 24 h, with the maximum reached at 8 h. At 7 days, the ratio of AST/ALT was slightly below the control (Figure 7). For all measurements, the Kruskall–Wallis test indicated significant dynamics compared to controls (*p* ≤ 0.0012), but no significance was found between the individual time points (Dunn’s multiple comparison test—*p* > 0.05).

#### 2.3.4. Liver Sections

Histologically, the liver was similar in both the control and treated groups and at all time points. Samples showed normal hepatic architecture, without evidence of toxic changes or inflammation (Figure 8).

**Figure 9:**
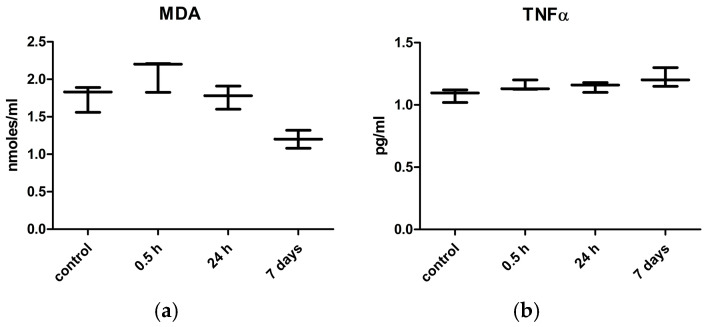
Measurement of (**a**) MDA and (**b**) TNFα from Wistar rat liver samples taken at different time points after one dose of KTZ-FUM (20 mg kg^−1^ bw), oral administration. Data represent the mean ± STDEV (n = 3).

To evaluate the possible protection against the oxidative stress and inflammation induced by the cocrystal in the liver tissue due to the Fumaric acid, we measured the MDA and TNFα at different time points (Figure 9) following the oral administration. In the liver, the data showed minimal oxidative lipid peroxidation, starting as soon as 30 min., and reversible within 24 h. This finding was combined with minimal changes in the pro-inflammatory cytokine TNFα, despite the persistence of KTZ-FUM in the bloodstream at this time point. For all measurements, the Kruskall–Wallis test indicated no significant dynamics compared to controls (*p* ≥ 0.05).

#### 2.3.5. Hematology and Biochemistry Measurements

Oral KTZ-FUM administration in Wistar rats led to an increase in the red blood cells and platelet counts as soon as 30 min, then the red blood cell counts slowly returned to normal at 72 h. The platelets number remained significantly higher compared to the control. Levels of cholesterol and glucose were not altered by the KTZ-FUM administration (Table 6).

#### 2.3.6. Epicutaneous Sensitization Test

**Figure 10:**
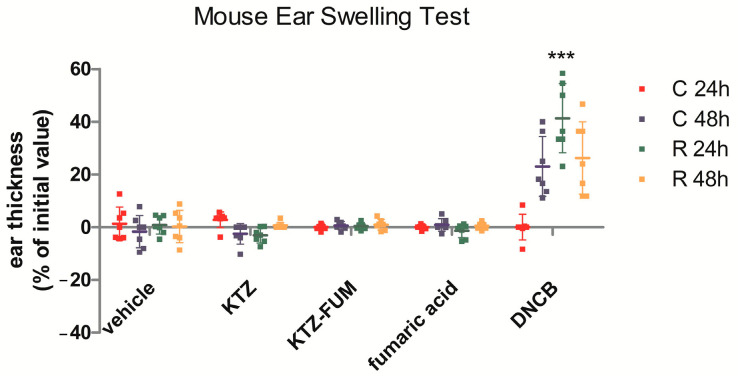
Mouse ear sensitization test. Comparative ear thickness in mice at different time points during the test, C 24 h—24 h after challenge, C 48 h—48 h after challenge, R 24 h—24 h after rechallenge, R 48 h—48 h after challenge; experimental groups: 1. negative control—represented by the vehicle, 2. KTZ, 3. KTZ-FUM, 4. FUM, and 5. positive control—represented by DNCB. Data are presented as mean ± STDEV (n = 5), *** = *p* ≤ 0.001.

We tested the epicutaneous sensitization potential of the cocrystal KTZ-FUM in comparison with the parent drug KTZ and Fumaric acid by using the mouse ear sensitization test (MEST). The positive control was represented by dinitrochlorobenzene (DNCB) and the negative control by the vehicle (70% ethylic alcohol). The positive control animals showed a high increase in ear thickness, starting at 48 h after challenge. This increase was maintained at rechallenge, with a maximum at R 24 h (Figure 10). In all the other groups, there was no alteration in ear thickness during the test. Two-way ANOVA showed significant time and treatment interaction between the groups (*p* < 0.0001).

**Figure 11:**
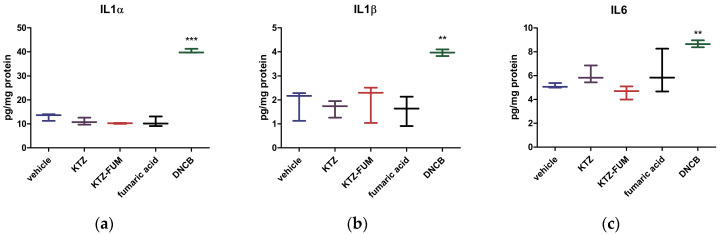
Inflammatory markers measured from the ear tissue collected at R48 h through ELISA (**a**) IL1α, (**b**) IL1β, and (**c**) IL6. Experimental groups: 1. negative control—represented by the vehicle, 2. KTZ, 3. KTZ-FUM, 4. FUM, and 5. positive control—represented by DNCB. Data are presented as mean ± STDEV (n = 3), ** = *p* ≤ 0.01, *** = *p* ≤ 0.001.

To further explore the inflammatory potential of the locally applied KTZ-FUM compared to the parent drug, we measured the inflammatory cytokines IL6 and IL1α and β in the treated ear tissue (Figure 11). As expected, the DNCB group showed a significant increase compared to the control of all cytokines. KTZ and FUM increased IL6, while IL1α and β were decreased, showing a low anti-inflammatory effect, although not significant. KTZ-FUM induced the decrease in IL6 and IL1α, showing anti-inflammatory potential. The Kruskall–Wallis test showed significance between groups for IL6 and IL1α (*p* ≤ 0.04) and not for IL1β (*p* = 0.09).

#### 2.3.7. Histopathology of Ear Sections

**Figure 12:**
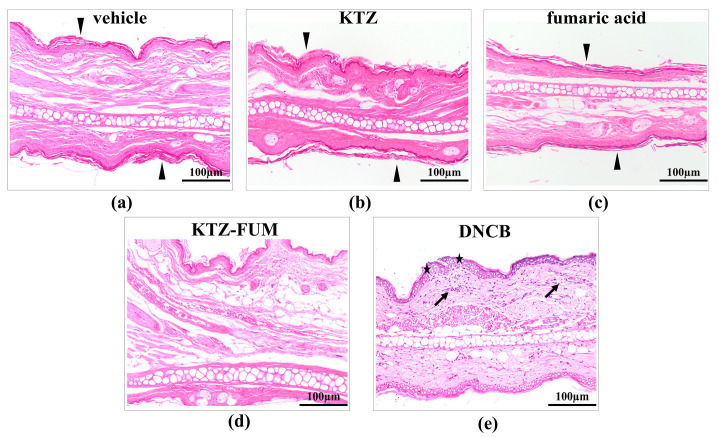
Histological analysis of ear pinna following the mouse ear sensitization test—(**a**) vehicle-negative control- (**b**) KTZ- (**c**) FUM-, and (**d**) KTZ-FUM-treated mice showed normal histological features, such as keratinized epithelium (arrowheads), and dermal structures (connective tissue, vessels, and adnexal structures) without any inflammatory changes; (**e**) DNCB-positive control. DNCB-treated mice exhibited contact dermatitis characterized by increased ear overall thickness, compared to vehicle and the other treated groups. The epithelium was multifocally irregularly thickened, with epidermal spongiosis (stars) in the basal and spinous layer. The dermis had mild, multifocal inflammatory infiltration with lymphocytes and plasma cells (arrows) and edema. HE stain. Bar = 100 μm.

In the DNCB-treated group, histological exam revealed contact dermatitis, with mo-derate inflammation, epidermal spongiosis in the basal and spinous layers, slight dermal edema, and multifocal inflammatory infiltration with lymphocytes and plasma cells. No histological changes were seen in the control–vehicle-treated group, KTZ, FUM, and in the KTZ-FUM-treated group (Figure 12).

### 2.4. Molecular Docking Study

The binding energies of KTZ, FUM, cocrystal, and posaconazole (original ligand) to the sterol 14α-demethylase enzyme from *Candida albicans* (PDB id: 5FSA) were predicted via molecular docking (Table 7).

Posaconazole, the original ligand, scored a binding energy of −12.08 ± 0.14 kcal mol^−1^. FUM enhanced KTZ’s binding energy by more than 1 kcal mol^−1^. Among all studied systems, the KTZ-FUM cocrystal–sterol 14α-demethylase complex exhibited the lowest binding energy (−12.54 ± 0.23 kcal mol^−1^). This value for the binding energy indicates that the cocrystal had the highest affinity within the tested systems.

**Figure 13:**
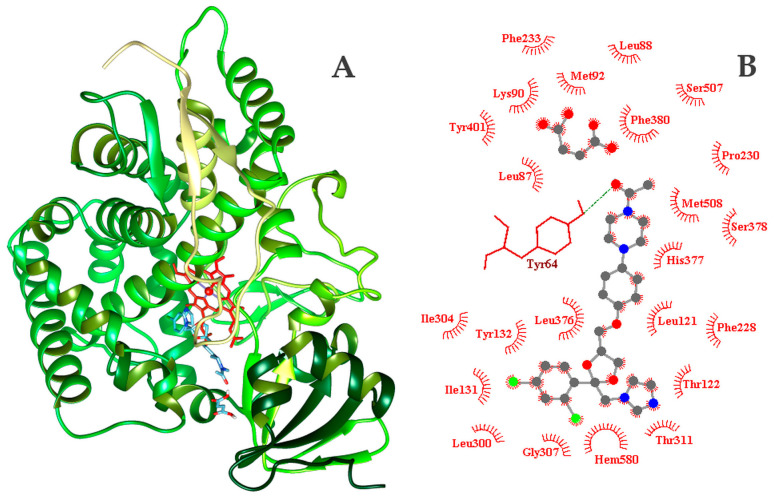
(**A**) Docked position of cocrystal (blue) to sterol 14α-demethylase from *Candida albicans* (PDB id:5FSA) together with (**B**) the 2D interaction diagram (ball-stick—cocrystal) and residues forming the binding site. HB is indicated by green dashed line. The red residues are involved in hydrophobic interactions.

The binding site of sterol 14α-demethylase, positioned in the interior of the macromolecule near the heme group, forms a pocket-like structure (Figure 13) that promotes the geometrical specificity of ligands to the detriment of other types of interactions. This site is defined by the following amino acids: LEU87, LEU88, LYS90, MET92, LEU121, THR122, ILE131, TYR 132, PHE228, PRO230, PHE233, LEU300, ILE304, GLY307, THR311, LEU376, HIS377, SER378, PHE380, SER507, TYR401, SER507, MET508.

The FUM coformer interacts with sterol 14α-demethylase through hydrogen bonds with the TYR118, HIS377, and SER378 residues through oxygen atoms, while hydrophobic interactions are formed with the LEU121, LEU376, and MET508 residues. KTZ forms a single hydrogen bond with TYR64 through its terminal oxygen atom, and it is involved in hydrophobic interactions with the following residues: LEU121, THR122, ILE131, TYR 132, PHE228, PHE233, LEU300, ILE304, GLY307, THR311, LEU376, HIS377, SER378, PHE380, SER507, MET508. The KTZ-FUM cocrystal exhibits the same hydrogen bond interaction as KTZ (with TYR64) and additionally forms hydrophobic interactions with several other residues, including LEU87, LEU88, LYS90, MET92, PRO230, TYR401, SER507, in addition to the above listed for KTZ.

#### Reactivity Descriptors Based on DFT

The parameters describing a compound’s reactivity, namely ionization potential (I), electron affinity (A), the HOMO-LUMO band gap (HLG), global hardness (η), global softness (σ), electronegativity (χ), chemical potential (μ), and the global electrophilicity index (ω), were also calculated for the KTZ, FUM, cocrystal, and original ligand, with results listed in Table 8.

Based on the HLG values in Table 8, KTZ is more reactive than posaconazole, the original antifungal in the complex with sterol 14α-demethylase. Its reactivity increases further in its cocrystal form, where the cocrystal has an HLG of 3.54 eV, indicating a shift from being more stable than the original ligand to being more reactive. The HLG difference between the cocrystal and posaconazole is 1 eV. FUM is the most stable compound among the four evaluated. The obtained HLG values of 3.54 eV and higher suggest that all compounds are stable. The electrophilicity index, which quantitatively measures a system’s ability to accept electrons, increases from posaconazole to FUM, indicating that posaconazole is less inclined to accept electrons, while FUM is more receptive to accept electrons from its environment. Additionally, KTZ’s electron-accepting capacity increases when in cocrystal form compared to its pure form.

## 3. Discussion

**Figure 14:**
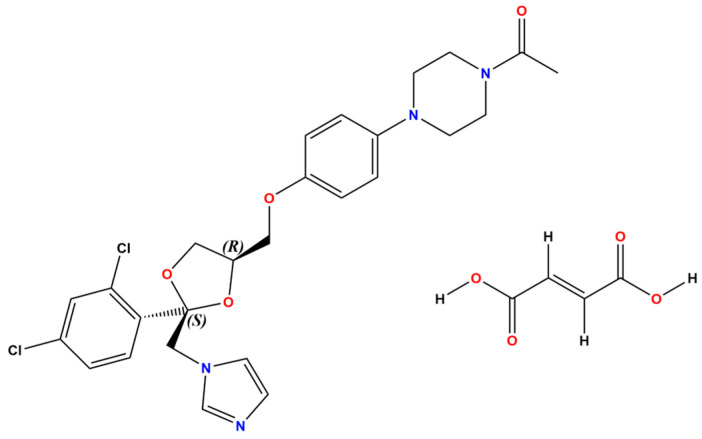
Chemical structure of Ketoconazole (KTZ) (**left**) and Fumaric acid (FUM) (**right**).

The focus for successful pharmaceutical drug development lies in the improvement of the physicochemical and pharmacokinetic properties of the API without compromising its pharmacological activity. Our previously formulated cocrystal between the antifungal BCS class II drug Ketoconazole and Fumaric acid (denoted KTZ-FUM, Figure 14) by crystal engineering resulted in an enhancement of the aqueous solubility (i.e., a 100-fold increase) and stability both in solid form and suspension as compared to the parent drug [21].

**Figure 15:**
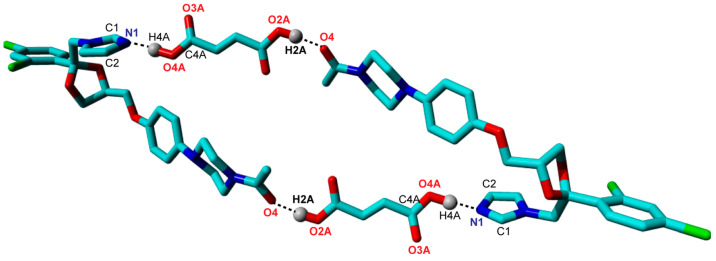
The four-member circuit network of KTZ-FUM cocrystal.

The KET-FUM cocrystal was prepared by two different approaches, the traditional solution crystallization method by solvent evaporation (i.e., acetone–water 8:2 (*V*/*V*) and acetone–methanol 1:1 (*V*/*V)*) and the versatile mechanochemical synthesis by ball milling, which involves the liquid-assisted grinding route (also known as solvent drop grinding, SDG) using drops of methanol. The crystal structure of the KTZ-FUM cocrystal (YINWAV code from Cambridge Structural Database [34]) contains one Ketoconazole and one Fumaric acid molecule in the asymmetric unit, connected via an O4···H2A−O2A hydrogen bond between the acetyl group of KTZ and one of the hydroxyl groups of FUM. The other hydroxyl group of FUM is also involved in a hydrogen bond with the imidazole group of another KTZ molecule, O4A−H4A···N1 (Figure 15). The nature of the cocrystal form is demonstrated by the H4A atom position (not transfer to N1) determined by single-crystal X-ray diffraction and confirmed by a few structural characteristics of interacting functional groups: (i) the C4A−O4 (1.284 Å) are longer bonds than C4A = O3A (1.204 Å), being specific to a neutral carboxylic group and (ii) the 105.69 deg value of the endocyclic C1-N1-C2 bond angle in the triazole ring of KTZ is specific to unprotonated nitrogen.

The stability of the cocrystal in our previous study was highlighted both in solution and solid state by (i) slurry experiments carried out in water and ethanol for at least 1 week, and (ii) accelerated testing, performed under storage conditions of 40 ± 2 °C/75 ± 5% RH for at least 4 months. The cocrystal dissociation to free KTZ form was not detected. Aiming to develop a rational and stable solid dosage form, we also reported its solid-state compatibility with acceptable pharmaceutical excipients in binary mixtures as part of the pre-formulation stage [35]. The results showed full compatibility of the KTZ-FUM with various excipients (i.e., hydroxypropyl methylcellulose K4M, lactose monohydrate, corn starch, silicon dioxide, and talc), thereby proving its capability to be marketed as a stable pharmaceutical cocrystal product.

### 3.1. Controlled Cocrystallization Process by Cooling

Developing pharmaceutical cocrystals implies a complex process that follows several distinctive phases within a regulatory framework established by the FDA [36]. A crucial part of the cocrystallization research is attributed to the nucleation and growth of cocrystals as a result of combining the coformers in a solution. In the cocrystallization process by cooling, the dissolution of both coformers is achieved by heating the solvent to a high temperature, followed by cooling, according to an established temperature profile. The solubility of the coformers decreases with decreasing solution temperature. This creates the supersaturation necessary for the occurrence of the nucleation process and crystal growth. Nucleation takes place only after the coformers solution has surpassed the metastable zone, defined as the zone between the concentration of solubility and the concentration of detection of the first nuclei (supersolubility) [37]. The metastable zone width—MSZW—is the temperature difference between the spontaneous nucleation temperature and the saturation one, and is affected by several variables, such as the cooling rate, stirring rate, or impurities [38]. Thus, defining the width of the metastable region for a compound is of utmost importance for the design and operation process at industrial scale. Scale-up development of the cocrystallization process is challenging because it must ensure reproducibility, purity, morphology and particle size, and finally a good yield of the product [39]. Another focus in cocrystals synthesis must be given to the screening stage for the identification of polymorphic forms. These forms can possess varying physicochemical properties, which can negatively affect the cocrystal’s performance [40].

Considering all mentioned above, a controlled cocrystallization process by cooling for the KTZ-FUM cocrystal was developed to determine the MSZW. Therefore, we established the optimal cocrystallization parameters to efficiently yield the cocrystal as a pure and single phase. Moreover, its reproducibility and stability in solution were also demonstrated. Among the seven tested solvent systems (e.g., acetone–water mixtures, ethanol–water 8:2 (*V*/*V*), 2-propanol) and a range of concentrations (10–160 mg mL^−1^), the measurement of MSZW for the optimal nucleation process was achieved just in the acetone–water 4:6 (*V*/*V*) mixture and ethanol. The highest cocrystallization yield was obtained in the case of a KTZ concentration of 100 mg mL^−1^ in the acetone–water 4:6 (*V*/*V*) solvent mixture.

### 3.2. Scale-Up Experiments of the Ketoconazol-Fumaric Acid Cocrystal

Up-scaling the production of pharmaceutical cocrystalline materials from laboratory small-scale screening (miligrams) and laboratory up-scaling (grams) to marketed drug products (kilo to multikilo) involves well-designed and established processes in order to provide high-purity cocrystals with high yields.

Two different laboratory up-scaling approaches, cocrystallization by cooling in solution and mechanochemistry synthesis (i.e., SDG), were employed for the KTZ-FUM cocrystal production at the scale of gram-size quantities according to cocrystallization parameters obtained within the MSZW. Solvent drop grinding is a commonly used, eco-friendly, and economically feasible approach for faster cocrystals production at large scale [41]. This method also enhances yields and accelerates reaction rates compared to solution-based procedures, and does not imply additional purification steps, e.g., liquid extraction.

Both methods successfully enabled the production of the pure KTZ-FUM cocrystal solid form to 1 g batch sizes with high yields, and without traces of residual pure components.

### 3.3. Biological Assays

#### 3.3.1. Cell Viability

In both cell lines, the dermal fibroblasts and hepatocarcinoma cells, KTZ-FUM was better tolerated than KTZ, showing improved biocompatibility in vitro. Fumaric acid inhibited cell viability. Liver toxicity of Fumaric acid esters (FAEs) was previously reported in psoriatic patients treated with these drugs and was not related to alcoholic abuse [42]. Fumaric acid esters were shown to inhibit inflammation by decreasing the differentiation of dendritic cells [43] and to inhibit neo angiogenesis by altering the metabolism of endothelial cells [44] through increased glycolysis and decreased oxidative metabolism, without compromising the cell viability even at a 100 µM concentration of dimethyl fumarate. Similar metabolic effects were found in MDA-MB 231 breast cancer cells and HGF-human gingival fibroblasts, but not in HeLa cancer cells [45]. In the HepG2 cell line, our viability results are similar to those obtained by others [46], using different FAEs.

#### 3.3.2. Oxidative Stress

Exposure of the cells to KTZ induced a lower MDA, as previously reported by us [47], and this effect was increased in the case of KTZ-FUM and decreased for Fumaric acid exposure. The antioxidant effects induced by Fumaric acid esters were shown to be dose-dependent for concentrations ranging between 0.1 mM and 3 mM on in vitro HepG2 cultures. The main antioxidant mechanism was due to the activation of the NRF2 signaling pathway [45]. The concentration of 6 µM used for the current experiment was much smaller, not enough to induce an antioxidant effect. However, the cocrystal showed improved antioxidant ability, when compared to the parent drug, suggesting a synergic effect of the KTZ and Fumaric acid even at this low concentration that might be beneficial for the therapy.

#### 3.3.3. Pharmaco-Dynamic Profile

Improving bioavailability has been the target of many new KTZ formulations, such as KTZ-loaded lipid nano formulations, solid lipid nanoparticles, and nanostructured lipid carriers. Even though Fumaric acid is a poorly water-soluble acid on its own, it may be of use with KTZ in granule form to promote a controlled release profile [48]. Studies on these formulations reported increased in vitro antifungal efficiency against *Candida albicans* and improved bioavailability in comparison to control KTZ suspensions when administered to Wistar rats [49]. With regards to efficacy, KTZ-loaded poly-lactic acid (PLA) nanoparticles were more effective against *Candida species* and dermatophytes than the free drug in vitro [50]. Hyaluronic acid-based gels with KTZ-loaded nanostructured lipid carriers showed improved drug diffusion and antifungal activity in vitro and ex vivo [51].

In a previous article, we demonstrated that the cocrystal obtained by using *p*-amino benzoic acid (PABA) and Ketoconazole (KTZ-PABA) showed an improved relative bioavailability of 6.72 over the KTZ in Wistar rats [47]. The data showed that the increased solubility of the KTZ-PABA led to a higher plasma concentration of 66.39 µg mL^−1^ at 1 h after the oral administration of 20 mg kg^−1^ bw. Although the maximum plasma concentration of the KTZ-PABA was higher than that of KTZ-FUM (52.21 µg mL^−1^), the relative bioavailability of the KTZ-FUM compared to KTZ was better. This is probably because of the increased solubility of the KTZ-FUM compared to the KTZ-PABA cocrystal and the more controlled release of the KTZ due to the presence of the Fumaric acid.

#### 3.3.4. Hepatotoxicity

Hepatotoxicity is one of the severe adverse effects of systemic KTZ-based therapy. In biopsy samples from 14 patients suffering from hepatic injury after KTZ treatment, centrilobular cholestasis or necrosis were reported, the latter being associated with primarily monocytic inflammatory infiltration [52]. Biochemically and histologically, hepatocellular necrosis is the most common type of hepatic injury observed in humans across studies [53].

In a study that compared the degree of hepatic injury in 66 Sprague Dawley rats that received oral antifungal agents (Fluconazole, Ketoconazole, Itraconazole, Terbinafine, and Griseofulvin), no macroscopic hepatic changes were observed in any group, whereas biochemical assays revealed that KTZ led to the highest ALT and AST levels. There was, however, a poor correlation between serum enzyme levels and histological examination, the latter revealing that fluconazole treatment caused the most severe damage compared to all groups [54].

In our experiments, the dynamics of the ALT and AST levels showed the highest increase in the AST/ALT ratio at 8 h, followed by a decrease below the control level at 7 days. The biochemical alterations were not accompanied by histological changes.

KTZ toxicity has also been linked to oxidative stress. In addition to lanosterol 14-α-demethylase inhibition, it has been suggested that fungicidal imidazoles owe a share of their effects to the generation of reactive oxygen species (ROS) in target pathogens [55,56,57]. Furthermore, in vitro murine [58] and human cell [59] study models demonstrated that, when exposed to Ketoconazole, healthy tissue is subject to oxidative stress, mitochondrial disfunction, and apoptosis. In a study on TM4 mouse Sertoli cells, pre-treatment with *N*-acetylcysteine mitigated cytokine production and ROS accumulation during azole treatment, and rescued the cells from apoptosis. This indicated that azole-induced cytotoxicity may be ROS-dependent [60]. For the investigation of KTZ-induced oxidative stress and pro-inflammatory activity, others have used different biomarkers, such as lactate dehydrogenase (LDH), superoxide dismutase (SOD), catalase (CAT), glutathione S-transferase (GST), malonealdehyde (MAD), and ascorbate peroxidase (APX) [61,62,63].

Studies investigating how to offset the KTZ-induced hepatotoxicity have shown that dietary supplements, such as sesame oil [64] and gentiana lutea [65], and hormones like melatonin [63] may reduce the hepatotoxic effects of KTZ as a result of their respective antioxidant effects.

In our experimental setting, the increase in MDA immediately after the oral administration was not significant, and it was followed by normalization within 24 h. This was combined with insignificant changes in the inflammatory cytokine TNFα.

Others showed, in a study on Wistar rats, that Fumaric acid co-administered with cadmium at hepatotoxic doses demonstrated a protective effect by anti-inflammatory and antioxidative mechanisms [66]. Rat hepatocytes showed increased DNA synthesis following Fumaric acid administration despite mitomycin C and aflatoxin B1 intoxication [67]. Liver and kidney damage incurred by Mitomycin C was also shown to decrease with concurrent administration of Fumaric acid [68]. Initial studies on Fumaric acid have affirmed its low toxicity in humans, with no evidence of adverse effects recorded for 75 subjects that received 500 mg daily for one year [69].

#### 3.3.5. Hematology and Biochemistry Measurements

Some of the less common reported side effects (≤1%) in humans during KTZ oral administration were thrombocytopenia [70], leukopenia, hemolytic anemia, and dyslipidemia [71]. In our experimental setting, the oral KTZ-FUM unique dose induced a temporary red blood cell count increase in the first 72 h and a slight thrombocytosis.

#### 3.3.6. Epicutaneous Sensitization Test

When used as a local treatment, KTZ may reduce inflammation. Applied topically on the skin of guinea pigs exposed to living or killed *Staphilococcus aureus*, KTZ exhibited a similar or improved anti-inflammatory efficacy in comparison to hydrocortisone acetate [72]. Similar results were obtained in a double-blind study investigating seborrheic dermatitis treatment in humans, which did not find statistically significant differences in symptom alleviation between hydrocortisone and KTZ treatments [73]. This suggested that the anti-inflammatory activity is part of the therapeutic benefit of the drug. One of the mechanisms by which KTZ directly decreases inflammation was proven to be lypooxygenase-5 inhibition, thus limiting the biosynthesis of leukotrienes [74]. KTZ can also reduce prostaglandin production via COX-2 down-regulation, a property which was exploited to trigger apoptosis via mitophagy in neoplasms such as epidermoid [75] and hepatocellular carcinoma [76], and to reduce drug resistance in human chronic myelogenous leukemia cells [77]. In previous studies, we found increased COX2 levels in vitro on human dermal fibroblasts and endothelial cells [47]. The same effect was found, although reduced, following local ear application on mouse ears, combined with increased anti-inflammatory cytokine IL10 [78]. When the cocrystal KTZ-PABA was used, the KTZ-induced increase in COX2 was decreased both in vitro and in vivo [78].

In tests for the evaluation of the skin sensitization potential by murine local lymph node assay and the guinea pig maximization test, FUM gave negative results [79], although its esters are contact allergens [80]. Topical KTZ use is linked to irritant contact dermatitis and potential sensitization [81], with rare reports of allergic [82] and, exceptionally, photoallergic contact dermatitis [83].

In our experiment, the KTZ-FUM and KTZ or Fumaric acid did not induce any allergic reactions following local applications. Moreover, the ear tissue showed a slight decrease in IL1α and IL 6 pro-inflammatory cytokines, indicating a reduced anti-inflammatory effect, as previously reported by us for the KTZ-PABA cocrystal [78] and others [63].

### 3.4. Molecular Docking Study

According to a former study, the in vitro antifungal activity of the KTZ-FUM cocrystal was evaluated on various fungal species (e.g., *Candida albicans*, *Trichophyton rubrum*) and revealed that KTZ therapeutic activity was not altered due to its formulation with the FUM coformer [84]. Considering these data, KTZ, FUM, cocrystal, and posaconazole (original ligand) were further evaluated in silico to determine the influence of FUM on KTZ’s affinity for the sterol 14-α demethylase (CYP51) enzyme from the pathogenic yeast *Candida albicans* (PDB id: 5FSA) and its binding energy.

Autodock Vina [85] is an automated routine used to predict ligand–receptor interactions. It performs a detailed scan across all available degrees of freedom as torsion angles of the ligand combine with a rapid grid-based energy evaluation. Through the application of a search algorithm and an empirical free energy scoring function, the routine refines the many possible docking conformations to identify the most probable one—the one with the lowest binding energy between the receptor and the ligand. As the algorithm is non-deterministic, each run generates stochastic results. Thus, for each ligand–receptor pair across the four systems (four ligands and one receptor), 10 binding modes were generated per run. The exhaustiveness parameter was set at its default value of 8. The code was run 20 times, producing 200 docking conformations for each system. Consistent with the methodology outlined in our previous work [86,87], this sample size was deemed enough to characterize the ligand–sterol 14α-demethylase interactions from both the perspective of predicted binding energy and geometrical specificity.

Among all studied systems, the KTZ-FUM cocrystal interacted more effectively with the enzyme, as the FUM coformer enhanced its binding affinity. This enhancement of KTZ affinity has been previously observed with *p*-hydroxy benzoic acid [88].

The highest occupied molecular orbital (HOMO) and the lowest unoccupied molecular orbital (LUMO) of a molecule are key indicators of its chemical reactivity and stability. The calculation of their energies is essential for understanding intermolecular interactions. The HOMO energy reflects a molecule’s electron-donating capability, while the LUMO energy represents its electron-accepting potential. According to Koopmens theory, the first ionization energy of a molecule is equal to the negative value of its HOMO energy [89]. Building on this theory, several parameters have been defined over time to provide a more detailed characterization of a compound’s reactivity, including ionization potential (I), electron affinity (A), the HOMO-LUMO band gap (HLG), global hardness (η), global softness (σ) [90], electronegativity (χ) [91], chemical potential (μ), and the global electrophilicity index (ω) [92,93]. The HLG describes the charge transfer interactions between a molecule and its surroundings, and its value represents the energy required to remove an electron from HOMO. A smaller HLG indicates a faster reaction and is a key reactivity descriptor of molecular stability. More reactive molecules exhibit smaller HLG values and higher softness values.

The molecular docking study reveals that KTZ, FUM, and the cocrystal interact with the sterol 14α-demethylase enzyme from *C. albicans* through a combination of hydrogen bonds and hydrophobic interactions. Moreover, the cocrystal exhibited greater chemical reactivity than the original ligand, posaconazole, as indicated by HLG and increased softness. Additionally, KTZ’s electron-accepting capacity increases in its cocrystal form, thus making it more reactive than the original ligand.

## 4. Materials and Methods

### 4.1. Materials and Reagents

Commercial form Ketoconazole was purchased from Melone Pharmaceutical Co., Ltd. (Dalian, China), and Fumaric acid from Merck KGaA, Darmstadt, Germany. Reagent-grade solvents ethanol, acetone, 2-propanol, dimethyl sulfoxide (cell culture grade) (Merck KGaA, Darmstadt, Germany), and ultrapure water (prepared by Milli-Q Ultrapure purification system, East Lyme, CT, USA) were used. Polyvinylpyrrolidone K30 (PVP K30), used as a suspending agent, was purchased from Tokyo Chemical Industry Co., Ltd., Tokyo, Japan. Ethyl acetate used for KTZ-FUM extraction from serum samples from Chimopar Trading SRL (Bucharest, Romania); HPLC-grade acetonitrile from VWR Chemicals (Rosny-sous-Bois, France) and formic acid from Crystal R Chim SRL (Bucharest, Romania). The solids and solvents were used as received without any further purification.

For in vitro and in vivo analysis, the following were employed: dermal fibroblasts (BJ-ATCC CRL-2522™) and hepatocarcinoma cells (HepG2-HB-8065, ATCC, Gaithersburg, MD, USA), Dulbecco’s modified Eagle medium (DMEM), fetal calf serum (FCS), gentamycin, amphotericin from Biochrom AG, Berlin, Germany; dimethylsulfoxide (DMSO) cell culture grade (Merck KGaA, Darmstadt, Germany); MTS assay—CellTiter 96^®^ AQueous Non-Radioactive Cell Proliferation Assay (MTS, Promega Corporation, Madison, WI, USA); malondialdehyde, Petri dishes (TPP, Transadingen, Switzerland); hematoxylin−eosin (HE) from Sigma-Aldrich Co, St. Louis, MO, USA; vacutainers (Vacuette Z serum clot activator tubes; Greiner Bio-One Ltd., Stonehouse, UK); Aspartate Aminotransferase (AST/GOT), Alanine Aminotransferase (ALT/GPT) Activity Assay Kits, Glucose (Glu) Colorimetric Assay Kit, Free Cholesterol (FC) Colorimetric Assay Kit and respectively Malondialdehyde Colorimetric Assay Kit (TBA Method) provided by ElabScience (Wuhan, China); soluble tumor necrosis factor alpha (TNF-α, Quantikine ELISA Immunoassay kit, R&D Systems, Inc., Minneapolis, MN, USA); vitamin A and dinitrochlorobenzene (Sigma-Aldrich, St. Louis, MO, USA); Freund’s complete adjuvant (Thermo Scientific™, Waltham, MA, USA); standard food for lab animals; rats were purchased from Cantacuzino Institute, Bucharest, Romania.

### 4.2. Cocrystallization Process by Cooling for Metastable Zone Width (MSZW) Determination

In 1.5 mL glass vials, known amounts of Ketoconazole and Fumaric acid in 1:1 molar ratio, and determined volume of solvent (0.5 or 1 mL) were added. A wide range of concentrations between 10 and 160 mg mL^−1^ and 7 solvent systems were tested: acetone–water mixture as 1:1, 9:1, and 4:6 (*V*/*V*) ratios, ethanol, ethanol–water 8:2 (*V*/*V*), 2-propanol, and 2-propanol–water 8:2 (*V*/*V*). The obtained suspensions were subjected to a controlled heating–cooling cycle performed on a magnetic stirrer (Heidolph, Scwabach, Germany). During the experiment, the vials were kept in a glycerin bath. The complete dissolution was reached under a controlled 1–3 °C min^−1^ heating rate and magnetic stirring; the solutions were then maintained at dissolution temperature for 5 min, followed by slow, controlled cooling, with 1–3 °C min^−1^ cooling rate to 10 °C, while the precipitation was completed. The solvents were removed by evaporation at room temperature until the solid materials were completely dry and were analyzed by PXRD.

### 4.3. Scale-Up Experiments of the Ketoconazole-Fumaric Acid Cocrystal

#### 4.3.1. Cocrystallization by Cooling in Solution

The scaling experiments by cooling cocrystallization method in solution were performed with the synthesizer process station Eyela PPS-CTRL 1 (Tokyo Rikakikai Co., Ltd., Tokyo, Japan), and denoted SU-E1 and SU-E2. The Eyela platform is equipped with 5 reaction vessels and enables individual setting of synthesis parameters, e.g., temperature, stirring. The reaction slurry mixtures composed of 1 g Ketoconazole, 0.218 g Fumaric acid, and the solvent, namely acetone–water mixture 4:6 (*V*/*V*) 13 mL and ethanol 20 mL, were homogenized by magnetically stirring at r.t. for 10 min and 400 rpm. The suspensions were heated to 65 °C under stirring (1000 rpm) with 2 °C min^−1^ heating rate until the solids fully dissolved. After the heating was stopped, the solutions were slowly cooled to 5 °C by 3 °C min^−1^ cooling rate. The solutions were kept at 5 °C for 24 h and then decanted. The residual solvents were removed by evaporation at room temperature until the materials were completely dry.

#### 4.3.2. Mechanochemical Synthesis

The mechanochemical scale-up experiments were carried out using a MM400 vibration ball milling (Retsch, Haan, Germany) and 5 mL stainless steel reaction jar by solvent drop grinding syntheses and are denoted SU-SDG1 and SU-SDG2. Equimolar quantities of pure components consisting of 1 g Ketoconazol and 0.218 g Fumaric acid were weighted followed by addition of 250 µL of solvent, i.e., acetone–water mixture 4:6 (*V*/*V*) or ethanol into the jar mill, which contained one stainless steel ball. The reaction mixtures were milled for 90 min at a frequency of 30 Hz. The resulting white powders were distributed on filter paper and air-dried.

### 4.4. Powder X-Ray Diffraction (PXRD) Characterization Method

KTZ-FUM cocrystal identity was confirmed by PXRD technique on a Bruker D8 Advance powder diffractometer using Cu Kα1 radiation (λ = 1.54056 Å). The *θ−2θ* Bragg−Brentano configuration geometry and incident beam Ge (111) monochromator were used. The measurements were performed at room temperature in the 3−40° range in steps of 0.02°.

### 4.5. In Vitro Assays

Dermal fibroblasts (BJ-ATCC CRL-2522™) and hepatocarcinoma cells (HepG2-HB-8065, ATCC, Gaithersburg, MD, USA) were employed. Cell culture medium used was Dulbecco modified Eagle medium (DMEM) supplemented with 5% FCS-fetal calf serum, 50 µg mL^−1^ gentamycin, and 5 ng mL^−1^ amphotericin, all from Biochrom AG (Berlin, Germany). Medium was changed twice a week. For all experiments, a medium with 2% FCS was used. To solve KTZ and KTZ-FUM in medium, substances were first solved in dimethyl sulfoxide at a concentration of 1 mg mL^−1^ to make a stock solution, and then the stock solutions were used to make the final concentrations in culture media immediately before the treatment. The final concentration of DMSO in the solutions added to the cells was lower than 0.05%, without negative viability effects [36].

#### 4.5.1. Cell Viability

Viability was measured using the MTS assay CellTiter 96^®^ AQueous Non-Radioactive Cell Proliferation Assay from (Promega Corporation, Madison, WI, USA), as indicated by the producer. Cells (BJ and HepG2) were cultivated on 96 well plaques at a density of 10^4^/well, accommodated in standard conditions for 24 h, then exposed to a fresh medium containing different KTZ, KTZ-FUM, and FUM concentrations for 24 h (range 0.01–100 µM). Negative controls were treated with medium. Viability was measured through colorimetry, using an ELISA plate reader (Tecan, Männedorf, Switzerland) at 540 nm. All experiments were performed in triplicate. Results are presented as % of untreated control, and the dose that caused a viability decrease below 70% was considered toxic. Inhibitory concentration (IC_50_) was calculated.

#### 4.5.2. Oxidative Stress-Induced Damage

Oxidative stress-induced damage was assessed in vitro by malondialdehyde (MDA) measurement from cell lysates. Cells (BJ or HepG2) were cultured on Petri dishes at a density of 10^5^/cm^2^ (TPP, Transadingen, Switzerland) for 24 h, then cells were exposed to 6 µM of each substance (KTZ, KTZ-FUM, Fumaric acid, respectively) for additional 24 h; afterwards, cells were washed and collected by scraping on ice. Cell lysates were then prepared as described [94]. The concentration of 6 µM was chosen to mimic the clinical scenario [47]. According to the reports by others, the plasma concentration of Ketoconazole after a therapeutic oral dose of 200 mg KTZ in healthy humans was 5.85 µM [95,96].

### 4.6. In Vivo Assays

#### 4.6.1. Experimental Design

The animal study protocol was approved by the Ethics Committee of the University of Medicine and Pharmacy Iuliu Hatieganu, Cluj-Napoca and the Veterinary Health Directorate, Romania (protocol code 329/17 08 2022).

Adult female Wistar rats, 3 months old (200 g body weight), were kept in standard cages type II, 5 animals/cage, in constant conditions of humidity (65%), temperature (21 °C), and day/night cycles of 12 h, fed with standard food (Cantacuzino Institute, Bucharest, Romania) and water ad libitum. The animals were randomly divided into 3 groups: controls—baseline, no therapy (3 animals), KTZ (33 animals), and KTZ-FUM (33 animals). Animals were administered through gavage a unique oral dose of 20 mg kg^−1^ body weight of KTZ or KTZ-FUM suspended in PVP K30 0.5% aqueous solution at a final concentration of KTZ—4 mg mL^−1^ as previously described [47]. The dose calculated according to Nair AB et al. corresponds to the clinically recommended oral dose of KTZ in humans of 200 mg/day [97]. The lab animals (3 animals/time interval) were humanely sacrificed at different time points following oral administration at 0.033 h, 0.083 h, 0.25 h, 1 h, 2 h, 4 h, 6 h, 8 h, 24 h, and 48 h, and 7 days [98]. Blood and liver samples were collected.

#### 4.6.2. Pharmacokinetic Profile

Serum KTZ and KTZ-FUM levels were measured by HPLC-MS. Regarding HPLC-MS Quantification, serum samples were processed according to the modified protocol described by Wang et al. [99]. According to this protocol, 0.5 mL of serum was extracted into 3 mL of ethyl acetate by shaking for 3 min, after which the mixture was centrifuged for 8 min at 4000 rpm. The organic layer, after being transferred to a clean tube, was evaporated at 37 °C under a stream of nitrogen. The residue was resuspended in 100 μL acetonitrile and then analyzed using a LC2010 Shimadzu (Shimadzu, Kyoto, Japan) high-performance liquid chromatograph, equipped with diode array (DAD) and quadrupole mass spectrometer (MS) detectors.

Chromatographic separation was performed on a Grace Alltima C18 column (100 × 3 mm, 3 μm), at a temperature of 40 °C, using acetonitrile-water mixture 1:1 (*V*/*V*) with 0.1% formic acid as the mobile phase. The elution was carried out isocratically with 0.4 mL min^−1^ flow rate. The injected sample volume was 10 μL. The mass spectrometer parameters were the capillary voltage of 1.5 kV, desolvation temperature of 250 °C, and interface temperature of 200 °C. The positive ionization mode was applied for KTZ mass spectra registration and negative ionization mode was applied for FUM mass spectra registration. For KTZ-FUM concentrations determination in the analyzed plasma, standard solutions of KTZ-FUM were prepared by successive dilutions starting from a concentration of 1 mg mL^−1^ in the concentration range of 0.001–0.1 mg mL^−1^.

KTZ-FUM from serum samples was quantified by external standard method using a calibration curve. The regression equation was expressed as y = 1 × 10^9^x + 4 × 10^6^ for KTZ and y = 3 × 10^6^x + 10,660 for FUM. The coefficient of correlation (R2) was about 0.9990 for all. Also, the limit of detection (LOD) and quantification (LOQ) were found to be 1.82 µg mL^−1^ and 3.61 µg mL^−1^ for KTZ and 1.664 µg mL^−1^ and 3.292 µg mL^−1^ for FUM.

Relative bioavailability (F) of the KTZ-FUM over KTZ, after a unique oral dose, was calculated using the following formula:

F = AUC KTZ-FUM/AUC KTZ × Dose KTZ/Dose KTZ-FUM,(1)

where AUC represents the area under the curve of serum concentration over time for KTZ, KTZ-FUM, respectively, determined by using GraphPad software.

#### 4.6.3. Hepatotoxicity Assessment

Liver transaminase activities were measured in the serum of the rats treated with KTZ-FUM at the same time points, using Aspartate Aminotransferase (AST/GOT) and Alanine Aminotransferase (ALT/GPT) Activity Assay Kits, respectively, both from ElabScience (Wuhan, China), as indicated by the manufacturer.

For the histopathological examination, the liver samples collected from the left lateral lobe and right medial lobe were fixed in 10% buffered neutral formalin, embedded in paraffin, cut at 4 µm thickness, and stained using the Hematoxylin–Eosin (H&E) staining method. The slides were examined under a BX51 Olympus microscope, and images were taken with an Olympus UC 30 digital camera (Olympus, Hamburg, Germany) and processed using the Olympus Stream Basic program.

Hematology and Biochemistry analyses: Hematology measurements were performed on freshly collected blood samples, in violet top vacutainers (K3-EDTA, 0.5 mL, Greiner Bio-One Ltd., Stonehouse, UK), using an automatic hematology Analyzer Mindray BC 6200, seria TW-1B001864, from Mindray, Shenzhen, China. For the biochemistry analysis of glucose and cholesterol from blood serum samples, the Glucose (Glu) Colorimetric Assay Kit and Free Cholesterol (FC) Colorimetric Assay Kit (both from ElabScience), respectively, were employed as indicated by the producer.

#### 4.6.4. Oxidative Stress and Inflammation

For the oxidative stress assessment, the level of malondialdehyde, a marker of lipid peroxidation, was measured from lysates of liver samples (in vivo) and cells-BJ and HepG2 (in vitro), using the Malondialdehyde Colorimetric Assay Kit (TBA Method) provided by ElabScience. Tissue sample homogenates (liver and ear) were prepared as previously described by David et al. [100]. The protein content in tissue homogenates was measured by Bradford method [101]. Inflammatory reaction was assessed by measurement of the tumor necrosis factor α (TNFα) in liver samples, using the rat TNFα ELISA kit from EIAAB. IL1α and β and IL6 measurement from the mice ear lysates were completed by ELISA, using the respective kits from Quantikine ELISA Immunoassay kit. Tissue homogenate samples were treated according to manufacturer’s instructions; readings were performed at 450 nm with correction wavelength set at 540 nm, using an ELISA plate reader (Tecan, Grödig, Austria).

#### 4.6.5. Mouse Epicutaneous Sensitization Test (MEST)

MEST was performed on BALBc mice (11 weeks old females) [102] purchased from Cantacuzino Institute (Bucharest, Romania). Mice were kept in constant conditions of humidity (65%), temperature (21 °C), and day/night cycles of 12 h, 5 animals/cage, fed with standard food (Cantacuzino Institute, Bucharest, Romania) and water ad libitum. Mice food was supplemented with vitamin A (250 UI g^−1^) to increase test sensitivity [103,104]. MEST protocol was performed as previously reported [78].

In total, 35 mice were randomly divided into five groups, 7 animals/group: group 1—control–vehicle (alcohol 70%), group 2—KTZ, group 3—KTZ-FUM, group 4—Fumaric acid, and group 5—dinitrochlorobenzene (DNCB, Sigma-Aldrich, St. Louis, MO, USA). On day one, under anesthesia, all mice received 30 µL/mouse of Freund’s complete adjuvant (Thermo Scientific™, Waltham, MA, USA), subcutaneously, in the right flank and the abdominal fur was carefully shaved, without skin abrasion. Then animals were treated with 100 µL of locally applied solution (abdominal skin) of each tested substance, in a concentration of 200 µg mL^−1^ solved in ethanol 70%, daily for the first 6 days [78,105]. The left ear thickness of each mouse was measured with an electronic caliper (initial value). Each mouse was treated with 50 µL/ear of the same solution on the left ear pinnae on day 7—challenge and on day 14—rechallenge. Ear thickness was measured at 24 h and 48 h after challenge and rechallenge, respectively [102]. Then mice were humanely sacrificed, under anesthesia, and ears were collected for inflammatory markers (IL1α, IL1β and IL6) assessment and histopathology.

Calculations were performed using the following formula:

% ear thickness = 100 × (A − B)/B,(2)

where A = ear thickness of the treated ear, B = initial ear thickness.

#### 4.6.6. Histopathology

For histopathological examination, the collected samples (liver and ears) were fixed in 10% buffered neutral formalin, embedded in paraffin, cut at 4 micrometer thickness, and stained using the Hematoxylin–Eosin (H&E) method. The slides were examined under a BX51 Olympus microscope, and images were taken with an Olympus UC 30 digital camera (Olympus, Hamburg, Germany) and processed using the Olympus Stream Basic program.

#### 4.6.7. Statistical Methods

The statistical difference between experimental groups was evaluated by nonparametric Kruskal–Wallis test, followed by Dunns post-test; the statistical significance of the treatments and time exposure within the experimental groups was tested using two-way ANOVA, followed by Bonferroni post-tests; results were considered significant for *p* < 0.05. For the in vivo pharmaco-dynamic profile of KTZ and KTZ-FUM, the AUC = area under curve was calculated. Statistical package used for data analysis was Prism version 4.00 for Windows, GraphPad Software, San Diego, CA, USA, www.graphpad.com. For IC_50_ calculation of the cell viability in vitro, the AAT Bioquest was used [106].

### 4.7. Molecular Docking Study Methods

The protein structure of sterol 14-α demethylase (CYP51) from the pathogenic yeast *Candida albicans* [PDB id: 5FSA] [107] was obtained from the Protein Data Bank and used as the receptor in this study. Prior to the building of the ligand–receptor systems, all water molecules, the original ligand, and other cofactors were removed from the receptor’s structure using Molegro Molecular Viewer 2.5 (Molexus Ivs, a CLC biocompany, Odder, Denmark). KTZ, FUM, and cocrystal were selected from the .cif file of our previously obtained structure [21]. KTZ, FUM, cocrystal, and the antifungal drug posaconazole originally complexed to the protein were selected as ligands. The geometries of all ligands were optimized prior to molecular docking in gas phase by using B3LYP hybrid exchange-correlation functional [108,109,110,111] coupled with 6-311+G(2d,p) (FUM and KTZ) or 6-311+G(d,p) (for cocrystal and original) basis sets as implemented in Gaussian software 16 Rev. C.01/C.02 [112]. The absence of imaginary frequencies for all ligands indicated that the resulted optimized geometries correspond to true minima. The ligand–receptor systems were built using Autodock Tools 1.5.6 [113], with only polar hydrogens added to the receptor structure, which was kept rigid. In contrast, the ligands were allowed full flexibility: 13 torsion angles were set for posaconazole, 4 for FUM, 8 for KTZ, and 14 for the KTZ-FUM cocrystal.

To ensure accurate binding of the studied ligands to the active site of sterol 14-α demethylase, the active site indicated by the original inhibitor in the crystallized complex was first validated. This was achieved by removing the original inhibitor from the crystallized ligand–receptor complex and performing a molecular docking scan using a search box that encompassed the entire receptor. The redocked original ligand was overlaid onto the crystallized complex, confirming the binding site position. Further, for the actual docking, the search box was centered at coordinates (x,y,z) = (192, 3, 38) of size 26 × 26 × 26 Å, and a grid spacing of 1 Å, containing the validated binding site. Finally, the ligands were docked to sterol 14-α demethylase using the Autodock Vina algorithm [85].

## 5. Conclusions

The focus for successful pharmaceutical drug development lies in the improvement of the physicochemical and pharmacokinetic properties of the API without compromising its pharmacological activity. The previously formulated cocrystal of the antifungal BCS class II drug Ketoconazole with Fumaric acid by crystal engineering resulted in an enhancement of the aqueous solubility (i.e., a 100-fold increase) and stability both in solid form and suspension as compared to the parent drug. Consequently, to evaluate the cocrystal antimycotic efficiency, the present work reports the successful development of both cocrystallization processes by cooling and mechanochemical synthesis. Furthermore, laboratory scale-up processes were carried out at 1 g batch size by both methods, and delivered the pure cocrystal in high yields of up to 90%.

Biological assays in vitro showed improved viability and oxidative-induced damage of the cocrystal over KTZ on human dermal fibroblasts and hepatocarcinoma cells; in vivo, on Wistar rats, the cocrystal increased oral KTZ bioavailability with transient minor biochemical increases in transaminases and without histological liver alterations. Locally applied on Balb C mice ears, the cocrystal induced no epicutaneuous sensitization and showed a slight anti-inflammatory effect.

Molecular docking showed that KTZ, FUM, and the cocrystal interact with the sterol 14α-demethylase (CYP51) enzyme from the pathogenic yeast *Candida albicans* through a combination of hydrogen bonds and hydrophobic interactions. Among all studied systems, the KTZ-FUM cocrystal interacted more effectively with the enzyme, and the FUM coformer enhanced its binding affinity. The cocrystal exhibited greater chemical reactivity than the original ligand posaconazole, as indicated by HLG and increased softness. Additionally, KTZ’s electron-accepting capacity increased in its cocrystal form, having an HLG of 3.54 eV, thus making it more reactive than the original ligand by 1 eV.

Overall, the cocrystal showed improved physical-chemical properties combined with enhanced biocompatibility, oral bioavailability, local anti-inflammatory effects, and predicted anti-fungal efficiency, which represent promising results for the future use of the KTZ-FUM cocrystal in clinical studies.

## Figures and Tables

**Table 1:** Cocrystallization experiments for MSZW determination.

Sample	[KTZ] (mg mL^−1^)	Solvent	Yield (%)
C1.1	60	acetone–water 4:6 (*V*/*V*)	74
C1.2	80		71
C1.3	100		85
C1.4	120		78
C2.1	40	ethanol	74
C2.2	50		77
C2.3	60		72
C2.4	70		75

**Table 2:** Scale-up conditions of KTZ-FUM cocrystal by cooling cocrystallization in solution.

Sample	Cooling Cocrystallization in Solution				
	Solvent/V (mL)	T_dis_ (°C) ^1^	T_pp_ (°C) ^2^	PXRD	Yield (%)
SU-E1	acetone–water 4:6 (*V*/*V*)/13	60	35	cocrystal	87
SU-E2	ethanol/18	62	31	cocrystal	89

^1^ dissolution temperature, ^2^ precipitation temperature.

**Table 3:** Scale-up conditions for KTZ-FUM cocrystal by mechanochemistry synthesis (SDG).

Sample	Solvent Drop Grinding		
	Solvent	V (µL)	PXRD
SU-SDG1	acetone–water 4:6 (*V*/*V*)	250	cocrystal
SU-SDG2	ethanol	250	cocrystal

**Table 4:** IC_50_ calculated for cultures of BJ dermal fibroblasts and HepG2 hepatocarcinoma cells subjected to various concentrations of KTZ, KTZ-FUM cocrystal, and FUM.

Cell Type	KTZ (µM)	KTZ-FUM (µM)	Fumaric Acid (µM)
BJ	7.45	42.39	7.26
HepG2	7.48	20.23	3.19

**Table 5:** Pharmaco-kinetic data of KTZ-FUM and KTZ in Wistar rats after one dose (20 mg kg^−1^ bw), oral administration.

	Unit	KTZ-FUM	KTZ
AUC_0–24h_^a^	μg h mL^−1^	2930.4 ± 0.1	228.96 ± 0.11
C_max_^b^	μg mL^−1^	52.213 ± 2.04	5.82 ± 0.7

^a^ area under serum concentration of each substance/time curve for 24 h, calculated using GraphPad software Prism version 4.00 for Windows; ^b^ maximum serum concentration of the drug.

**Table 6:** Hematology and biochemistry assessment following oral KTZ-FUM administration.

	RBC	Hb	HTC	WBC	PLT	CST	Glucose
	(×10^6^)	(g dL^−1^)	(%)	(×10^3^)	(×10^3^)	(mg dL^−1^)	(mg dL^−1^)
CTRL	7.62 ± 0.41	13.90 ± 0.41	41.30 ± 0.84	10.95 ± 0.40	580.01 ± 15.56	35.3 ± 0.53	80.69 ± 5.34
0.5 h	8.27 ± 0.92 *	15.9 ± 0.867	49.63 ± 2.82	9.17 ± 2.72	687.66 ± 47.24 *	33.35 ± 3.69	82.77 ± 0.28
24 h	8.22 ± 0.54	15.9 ± 0.1 *	48.03 ± 0.11	7.91 ± 2.89	747 ± 234.78	35.45 ± 2.26	82.9 ± 7.47
72 h	7.4 ± 0.62	14.26 ± 1.41	44.73 ± 3.91	9.09 ± 4.68	753.33 ± 45.05 *	31.95 ± 2.07	81.56 ± 4.65

RBC—red blood cells, Hb—hemoglobin, HTC—hematocrit, WBC—white blood cells, PLT—platelets, CST—total cholesterol; data are presented as mean ± STDEV, n = 3 for each measurement; statistical analysis was performed using Student TTEST, * = *p* < 0.05.

**Table 7:** Binding affinities of KTZ, FUM, KTZ-FUM cocrystal, and posaconazole to 14-α demethylase from *Candida albicans* (PDB id: 5FSA).

kcal mol^−1^	KTZ	FUM	Cocrystal	Original
average E_bind_	−11.36	−4.50	−12.54	−12.08
SD	0.31	0.00	0.23	0.14
min	−11.70	−4.50	−12.80	−12.30
max	−10.10	−4.50	−11.60	−11.80

Binding affinities expressed as average values for the best ligand of the 20 code runs together with the standard deviation (SD), as well as the min and max values obtained within the 20 code runs.

**Table 8:** Quantum chemical reactivity descriptors obtained on the optimized geometries of the selected ligands by Density Functional Theory calculations in gas phase at B3LYP/6-311+G(2d,p) (KTZ, FUM) or B3LYP/6-311+G(d,p) (cocrystal, original) level of theory.

eV	FUM	KTZ	Cocrystal	Original
E_HOMO_	−8.018	−5.542	−5.554	−5.760
E_LUMO_	−2.540	−1.488	−2.012	−1.160
I	8.018	5.542	5.554	5.760
A	2.540	1.488	2.012	1.160
HLG	5.478	4.053	3.542	4.600
η	2.739	2.027	1.771	2.300
σ	0.365	0.493	0.565	0.435
χ	5.279	3.515	3.783	3.460
μ	−5.279	−3.515	−3.783	−3.460
ω	5.088	3.048	4.041	2.602

I—ionization potential; A—electron affinity; HLG—HOMO-LUMO gap; η—global hardness; σ—global softness; χ—electronegativity; μ—chemical potential; ω—global electrophilicity index.

## Data Availability

The original contributions presented in this study are included in the article; further inquiries can be directed to the corresponding authors.

## Supplementary Materials

The following supporting information can be downloaded at: https://www.mdpi.com/article/10.3390/ijms252413346/s1.
