# Supplementary material for: Ketoconazole-Fumaric Acid Pharmaceutical Cocrystal: From Formulation Design for Bioavailability Improvement to Biocompatibility Testing and Antifungal Efficacy Evaluation
Source: Int J Mol Sci. 2024 Dec 12;25(24):13346. doi: 10.3390/ijms252413346 (PMC11678873; doi:10.3390/ijms252413346)
Supplement: Supplementary file 1 [file ijms-25-13346-s001.zip › Table S1.pdf]

Table S1. Cocrystallization experiments for MSZW determination

**Sample**	**[KTZ] (mg mL^{-1})**	**Solvent / mL**	**$T_{dissolution}$ (°C)**	**Obs.**	**$T_{precipitation}$ (°C)**
C3.1	30	acetone:water 9:1 (*V*/*V*) / 0.5	50	Clear, light brown solution	<5
C3.2	40		53		<5
C3.3	50		58		<5
C3.4	60		62		<5
C3.5	70		64		24
C4.1	5	acetone:water 1:1 (*V*/*V*) / 0.5	59	Clear, light brown solution	<5
C4.2	10		62		<5
C4.3	20		64		<5
C5.1	15	2-propanol / 1	60	Clear solution	<5
C5.2	20		62		<5
C5.3	25		64		<5
C5.4	30		66		17
C5.5	35		68		33
C6.1	15	2-propanol:water 8:2 (*V*/*V*) / 1	27	Clear solution	-
C6.2	20		28		-
C6.3	25		46		-
C6.4	30		48		<5
C6.5	35		51		<5
