# Supplementary material for: Ketoconazole-Fumaric Acid Pharmaceutical Cocrystal: From Formulation Design for Bioavailability Improvement to Biocompatibility Testing and Antifungal Efficacy Evaluation
Source: Int J Mol Sci. 2024 Dec 12;25(24):13346. doi: 10.3390/ijms252413346 (PMC11678873; doi:10.3390/ijms252413346)
Supplement: Supplementary file 1 [file ijms-25-13346-s001.zip › Table S2.pdf]

Table S2. Mechanochemistry synthesis (SDG) for KTZ-FUM cocrystal based 100 mg KTZ

Sample	Solvent Drop Grinding		
	**Solvent**	**V (μL)**	**PXRD**
SDG1- 100 mg	acetone:water 4:6 (*V*/*V*)	20	cocrystal
SDG2 -100 mg	ethanol	20	cocrystal
