# Supplementary figures and images for: Ketoconazole-Fumaric Acid Pharmaceutical Cocrystal: From Formulation Design for Bioavailability Improvement to Biocompatibility Testing and Antifungal Efficacy Evaluation
Source: Int J Mol Sci. 2024 Dec 12;25(24):13346. doi: 10.3390/ijms252413346 (PMC11678873; doi:10.3390/ijms252413346)

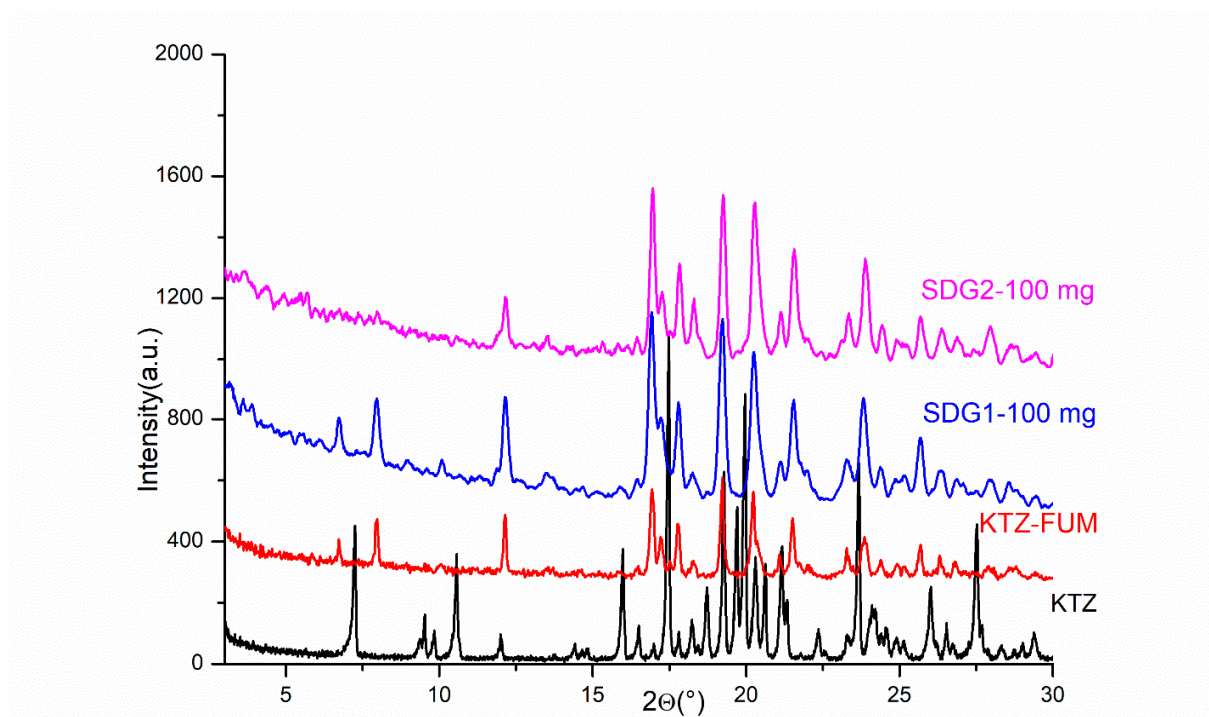

**Figure S1.** PXRD patterns for KTZ-FUM of SDG cocrystallization experiments based 100 mg KTZ

Supplement: Supplementary file 1 [file ijms-25-13346-s001.zip › Figure S1.pdf]
